# Trends in Diet Quality and Increasing Inadequacies of Micronutrients Vitamin C, Vitamin B12, Iron and Potassium in US Type 2 Diabetic Adults

**DOI:** 10.3390/nu15081980

**Published:** 2023-04-20

**Authors:** Hongbing Sun, Connie M. Weaver

**Affiliations:** 1 Department of Earth and Chemical Sciences, Rider University, 2083 Lawrenceville Road, Lawrenceville, NJ 08648, USA; 2 School of Exercise and Nutritional Sciences, San Diego State University, 5500 Campanile Drive, San Diego, CA 92182, USA; cmweaver@sdsu.edu

**Keywords:** type 2 diabetes, diet quality, HEI-2015, usual intake, increasing inadequacies of vitamins C, vitamin B12, iron and potassium

## Abstract

BACKGROUND: Prevalence of diabetes was high and rose significantly in the US between 1999 and 2018. A healthy dietary pattern that provides micronutrient adequacy is one of the most important lifestyle choices for battling the progress of diabetes. Yet, the patterns and trends of diet quality of the US type 2 diabetes are understudied. OBJECTIVES: We aim to examine the patterns and trends of diet quality and major food sources of macronutrients of US type 2 diabetic adults. METHODS: The 24 h dietary recalls of 7789 type 2 diabetic adults, comprising 94.3% of total adults with diabetes from the US National Health and Nutrition Examination Survey cycles (1999–2018), were analyzed. Diet quality was measured by the total Healthy Eating Index (HEI)-2015 scores and 13 individual components. Trends of usual intakes of vitamin C (VC), vitamin B12 (VB12), iron, and potassium and supplements from two 24 h recalls were also examined for type 2 diabetic population. RESULTS: Diet quality of type 2 diabetic adults worsened between 1999 and 2018 while that of US adults of general population improved based on the total HEI 2015 scores. For people with type 2 diabetes, consumption of saturated fat and added sugar increased while consumption of vegetables and fruits declined significantly, although consumption of refined grain declined and consumption of seafood and plant protein increased significantly. In addition, usual intakes of micronutrients VC, VB12, iron, and potassium from food sources declined significantly during this period. CONCLUSIONS: Diet quality generally worsened for US type 2 diabetic adults between 1999 and 2018. Declining consumptions of fruits, vegetables, and non-poultry meat may have contributed to the increasing inadequacies of VC, VB12, iron, and potassium in the US type 2 diabetic adults.

## 1. Introduction

Prevalence of diabetes and related comorbidities in the United States (US) rose significantly from 1999 to 2018 [1]. Approximately 10.5% and 34.5% of the US population aged 18 years and older had diabetes and pre-diabetes, respectively, in 2018 [2]. A crude estimation of type 2 diabetes accounts for about 94.8% (or 5.2% type 1) of the total incidence of diabetes among US adults aged 20 years and older in 2018 [2]. Uncontrolled diabetes can increase the risk of mortality from cardiovascular disease and disability-adjusted life-year loss [3].

Nutritional management remains one of the most important cornerstones in the prevention and management of type 2 diabetes because diet affects levels of serum glucose [4,5,6]. Although overall diet quality has been reported to be on the rise in the US adults [7], variations in trends of diet quality of food components exist. Diet quality also varied by age, social economic and racial groups, and time periods [8,9]. Recent changes in diet quality and individual components across the type 2 diabetic population remain understudied [4]. Evidence is limited in recent trends of macronutrient and food sources for type 2 diabetic population. Because total energy intake of a person during a period of time is relatively stable, change in consumption of one macronutrient component can lead to adjustment in another [7]. Changes in economy and nutrition-related policies, and new understanding of nutrition on glucose control, food processing methods, and culture over time, can also result in the shift of diet quality in diabetic population [7,9].

Using data from 10 consecutive cycles of the National Health and Nutrition Examination Survey (NHANES), the primary aim of this study was to investigate trends in diet quality and in food sources of dietary macronutrient intake among the US type 2 diabetic adults from 1999 to 2018. The secondary aim of this study was to examine the impact of changing diet quality on the intake adequacies of critical nutrients among the US type 2 diabetic adults, vitamin C (VC), vitamin B12 (VB12), iron, and potassium. The four nutrients were selected because intakes of VC, iron, and potassium were on the decline in the general population [10,11,12] and increasing use of common glucose control medication metformin [13] may lower VB12 in diabetes [14]. Low serum VC is related to increased risk of comorbidities and mortality in diabetic people [15,16]. Iron deficiency anemia is more prevalent among diabetic population because of their reduced iron absorption [17]. Declining potassium intake and increased hypokalemia can increase the risk for hypertension and cardiovascular diseases, while both are common in diabetic population [18]. In addition, VB12 deficiency could lead to hyperhomocysteinemia and increase the risk of macrovascular complications in people with type 2 diabetes [19]. We hypothesized that diet quality of US adults with type 2 diabetes is different from that of general population because of the dietary requirement for glucose control in the diabetic population, and the changing trends of diet quality can affect the adequacy levels of the above-mentioned nutrients disproportionally [20]. Evaluation of diet quality is important in identifying challenges and opportunities in diabetes control and can have policy and clinical implications for improving the management efficacy of US type 2 diabetic adults.

## 2. Methods

### 2.1. Study Design and Data

NHANES is a nationally representative cross-sectional study conducted by the National Center for Health Statistics (NCHS) of US Centers for Diseases Control and Prevention (US CDC). It collects information on health and nutritional status of the non-institutionalized civilian population in the US [21]. The study design, protocol, and data collection methods have been reported extensively elsewhere. More detailed information of the design and methods of NHANES is available on the NHANES website [22]. The NHANES study protocol was approved by the research ethics review board of the NCHS of US CDC, and all participants provided written informed consent [23]. Because NHANES data are publicly available, ethical approval for the analyses of the data in the current study is not needed.

**Figure 1:**
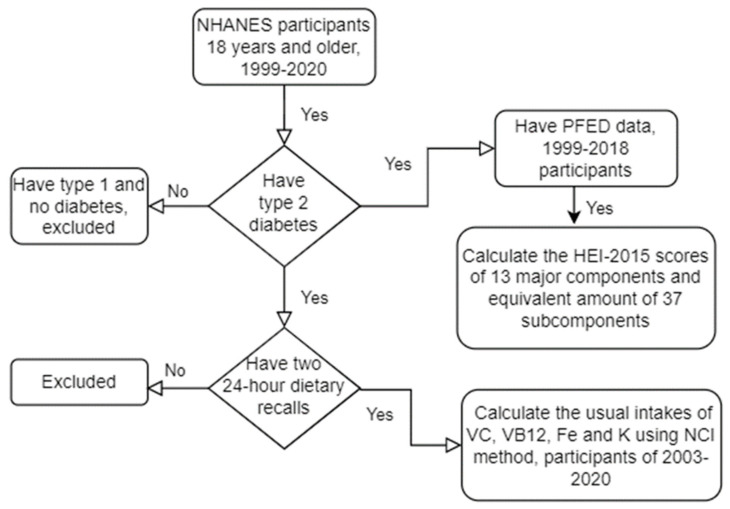
Flowchart of the study design.

All participants that were 18 years and older who had diabetes profile and dietary data during the NHANES survey between 1999 and 2018 were screened for this study (Figure 1) [21]. Following examples of prior studies, participants were classified as having diabetes by self-report, results of random glucose test ≥ 200 mg/dL, fasting glucose ≥ 126 mg/dL, and HbA1c ≥ 6.5%, or use of a glucose lowering medication other than metformin including a sulfonylurea, insulin, or incretin mimetic [9,24]. Metformin use alone was not considered diagnostic because of its possible consumption in the pre-diabetic population. The measures of insulin intake status, duration, age of diabetes diagnosis, and time difference between the diagnosis and insulin intake were used to separate the type 1 and type 2 diabetes following the treatment algorithm of Mosslemi et al. [25] and principles established in previous studies [26]. Information related to the race and prescription drugs were self-reported by NHANES participants according to categories provided by the NHANES. Five racial categories described in this study are Mexican American, other Hispanic, non-Hispanic white, non-Hispanic black, and other. Estimated glomerular filtration rates (eGFR) were calculated from serum creatinine and urinary albumin-to-creatinine ratio, using the Chronic Kidney Disease Epidemiology Collaboration PI equation (CKD-EPI-2009) [27]. Dietary data of only those identified as having type 2 diabetes were used in the diet quality and usual intake analyses (Figure 1).

### 2.2. Dietary Interview and Food Groups

NHANES nutritional assessment from the 24 h dietary recall interview includes nutrients and non-nutrient components from foods and beverages that were consumed during the 24 h period prior to the interview (midnight to midnight). Beginning in 2003, a second 24 h recall was also administered by NHANES via telephone interview approximately 3 to 10 days after the first recall [21].

The same definitions of food components used by NHANES were used in the US Department of Agriculture’s (USDA) Food Patterns Equivalents Database (FPED, 2005–2018) and MyPyramid Equivalents Database (1999–2004) across survey cycles for assessing the intakes of major food groups. Nutrients were evaluated based on year-cycle-specific versions of the USDA Food and Nutrition Database for Dietary Studies. Details on the component definitions can be found on the USDA FPED’s document files online [28]. Moreover, 2017–2018 are the most recent years that the USDA has the FPED needed for the calculation of the Healthy Eating Index (HEI)-2015 scores [28]. FPED converts the foods and beverages in the Food and Nutrient Database for Dietary Studies of NHANES into the 37 USDA Food Patterns components.

Usual intakes of nutrients are the preferred estimations of the averaged long-term adequacy of nutrient intakes [29]. For the usual intake analyses of VC, VB12, iron, and potassium, their dietary intakes and supplements of qualified participants were all obtained from two 24 h dietary recalls in this study. Because the second day intake data were only available after 2003–2004, analyses of usual intake of the four micronutrients were only performed for years of 2003–2004 and after (Figure 1). The analyses were extended into 2017–2020 for nine cycles because of the additional available data for analyses of usual intake in the 2017–2020 cycles.

### 2.3. Outcomes

The main outcomes of this study were the total Healthy Eating Index (HEI)-2015 score, HEI-2015 scores of 13 major food-category components, and the equivalent amount of the 37 subcomponents that were used to calculate the HEI scores. HEI-2015 is the latest version of the HEI that is commonly used to assess how well dietary components align with the USDA 2015–2020 Dietary Guidelines for Americans [30,31,32,33] and the overall diet quality. The total HEI-2015 score ranges from 0 (lowest) to 100 (highest). For the 13 components, 9 are adequacy components (total vegetables, greens and beans, total fruits, whole fruits, whole grains, total dairy, total protein foods, seafood and plant proteins, and fatty acids) and 4 are moderation components (refined grains, sodium, added sugar, and saturated fats). For the adequacy components, higher scores indicate higher intakes because higher intakes are more desirable, while for the moderation components, higher scores indicate lower intakes because lower intakes are more desirable [32]. The HEI-2015 scores were calculated using the population ratio method recommended by the National Cancer Institute (NCI) and USDA [30,32]. Their scoring standards are given in the Supplementary Table S1. Mean equivalent amounts of 37 subcomponents used to calculate the HEI scores of 13 components included whole fruits, total fruits, tomatoes and products, white potatoes, dark green vegetables, total vegetables, seafood high and low in n-3 fatty acids, meat, poultry, total meat, legume, milk, cheese, eggs, fatty acids, added sugar, oils, solid fats, etc. Definitions including the food sources constituting most of these subtypes are given in Table S2 in the Supplementary Materials and a full list can be found on the USDA website [34].

Additional outcomes of this study included the usual intakes of VC, VB12, iron, and potassium from the years 2003 to 2020 and their trends of inadequacy. The NCI method [35] was used to estimate usual intake based on the first and the second day intake data. The “SIMPLE” macro codes developed by Luo et al. [36] were incorporated into the pre- and post-processing of the NCI model to facilitate the calculation of usual intake. The first step of NCI method modeled the probability of consuming a given nutrient and the amount for nutrients that are not consumed daily by most persons. The second step involved estimating usual intake with parameters estimated from the first step using mixed effect linear regression on a transformed scale with a person-specific effect.

### 2.4. Statistical Analysis

The complex, multistage probability sampling design of the NHANES using the sampling weight, strata, and primary sampling unit parameters were applied in the analyses to obtain nationally representative estimates. For estimation of the statistical significance of differences in diet scores over time, a survey-weighted linear regression model was used to evaluate multiple regression terms using survey year as a continuous variable and age, race, and gender as covariates. The percentage changes were calculated using the differences of the means of modeled population in 2017–2018 and 1999–2000 over the means of 1999–2000. The percentage of energy of saturated fat was computed as proportion of energy from the saturated fat over total energy intake and is equal to (total saturated fat [g] × 9 [kcal/g])/total energy intake (kcal) × 100.

The means of demographic data, A1C, BMI, and eGFR were calculated using the Survey Analyses of Stata (version 17.0, StataCorp LLC). The HEI-2015 scores, the usual intake of nutrients, and other statistical results were calculated using SAS (SAS Institute Inc., version 9.4). Two-sided tests with a 95% significance level were conducted. Because there were no adjustments made for multiple comparisons in this study, the findings of analyses are interpreted as exploratory.

## 3. Results

### 3.1. Characteristics of Participants

A total of 7789 adults (4023 men and 3766 women), mean age of 59.2 (95% CI 58.7–59.7) years (Table 1), were identified as likely having type 2 diabetes between 1999 and 2018. A total of 580 participants identified as likely having type 1 diabetes using the treatment algorithm by Mosslemi et al. [25] were excluded. From 1999 to 2018, type 2 diabetes was 94.3% of total diabetes (i.e., type 1 diabetes 5.7%). The percentage of type 2 diabetes as the percentage of total population increased from 7.1% to 13.5% between 1999 and 2018. Mean BMI and eGFR increased significantly with the years after adjusted for age, race, and gender, while mean A1C level did not. All participants completed at least the first valid 24 h dietary recall and 87.1% completed the secondary dietary recall between 2003 and 2020. There were 1359 type 2 and 81 type 1 diabetic adults in 2017–2020 cycles and type 2 diabetes was 94.8% of the total diabetes.

From 1999 to 2018, the proportion of non-Hispanic white individuals with type 2 diabetes among all type 2 diabetes decreased from 62.2% to 58.9% in 2017–2018 and to 57.9% in 2017–2020, while the proportional share of other races with type 2 diabetes increased.

### 3.2. Trends in Diet Quality

**Figure 2:**
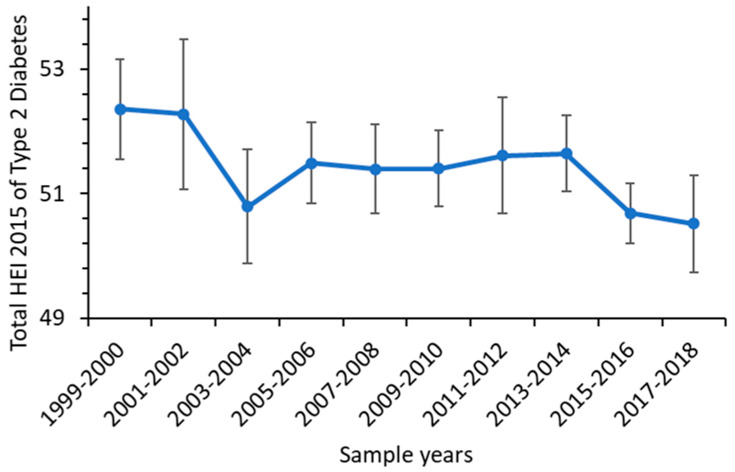
Total HEI-2015 scores of US type 2 diabetic adults from 1999–2000 to 2017–2018.

**Figure 3:**
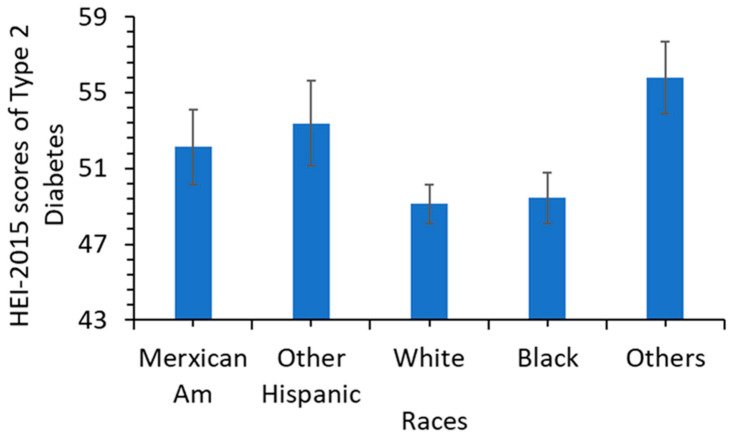
Total HEI-2015 scores of 5 races of US type 2 diabetic adults for 2017–2018.

From 1999 to 2018, overall diet quality decreased significantly for people with type 2 diabetes based on the total HEI-2015 score (Figure 2, Table 2). The mean total HEI-2015 score decreased from 52.35 to 50.52 (a decrease of 3.5%; *p* < 0.02). Among the races, diet quality as measured by HEI-2015 score was lower in non-Hispanic white and black diabetic adults than in other races (Figure 3).

Statistically significant changes occurred among 8 of the 13 individual components of the HEI-2015 diet score (Table 2) for type 2 diabetic adults from 1999 to 2018. The trends of HEI scores of some components were unsettling. Mean HEI scores of total fruits, whole fruits, and vegetables decreased by 20.2%, 29.3%, and 11.0%, respectively, for type 2 diabetic adults between 1999–2000 and 2017–2018. Mean HEI scores of added sugar and saturated fat decreased by 4.5% and 10.6%, respectively. For the added sugar and saturated fat, decrease in their HEI scores indicates an increase in their consumption because they are the moderation components.

Some changes in dietary patterns were positive. The HEI score of refined grain increased significantly by 7.0% between 1999–2000 and 2017–2018, indicating a significant decline in its consumption. Mean HEI score of seafood and plant protein consumption increased significantly by 39.7% between 1999–2000 and 2017–2018. Changes of the HEI scores of other five components between 1999 and 2018 were not significant.

#### 3.2.1. Trends in the 37 Dietary Subcomponents/Categories and Total Energy

From 1999 to 2018, consumption of total energy (sum of energy from fat, carbohydrate, and proteins) increased (*p* = 0.058) for type 2 diabetic adults. The trends of food categories of whole fruit, total fruits, total vegetable, whole grain, refined grain, and solid fat were all similar to their corresponding trends in HEI scores as expected (Table 3). In the meat subcategories, consumption of non-poultry meat decreased while consumption of poultry meat increased from 1999 to 2018, though not statistically significant. In dairy consumption, milk consumption decreased by 34.9%, while cheese consumption increased by 49.9% between 1999–2000 and 2017–2018. Significant increases in the consumption of oil and added sugar were also noticeable from 1999 to 2018 for the type 2 diabetic adults. Consumption of nuts and seeds increased significantly as well. Consumption of saturated fat rose to about 11.6% of the total energy consumption by 2017–2018.

#### 3.2.2. Trends of Usual Intakes of VC, VB12, Iron and Potassium

From 2003 to 2020, medians of usual intakes of dietary alone and total of dietary and supplement intakes of VC, iron, and potassium all declined significantly for type 2 diabetic adults. However, the median usual intake of dietary only for VB12 declined significantly, while the median usual intake of total dietary and supplement VB12 increased significantly for type 2 diabetic adults (Table 4). Contributions of supplementary intakes to their corresponding total dietary usual intakes were much higher for VC and VB12 than for iron and potassium. Percentages of total usual intakes of VC, VB12, and iron not meeting their corresponding estimated average requirement (EAR) and the total usual intake of potassium below its adequate intake level (potassium does not have an EAR) increased significantly for the type 2 diabetic adults in the US.

## 4. Discussion

From 1999 to 2018, overall diet quality represented by the HEI-2015 score worsened for type 2 diabetic adults in US, contrary to the rising trend (~2%, *p* < 0.01) of the general adult US population [7]. Though this decline in the total HEI-2015 score was relatively small (3.5%) and may be of limited importance, deterioration in consumption of total fruit, vegetables, saturated fat, and added sugar may be of clinical significance (Table 2). Improvement occurred in the decreased consumption of refined grain and increased consumption of seafood and plant protein. New scientific evidence and improved dietary guidelines on benefits of healthy diets might have helped the decline in the consumption of refined grain in the type 2 diabetic adults [32,37]. However, refined grain and saturated fats were still a large portion of the diet in the US type 2 diabetic adults. In addition, US type 2 diabetic adults still consumed a higher proportion of energy intake from low-quality carbohydrates represented by refined grains, fruit juice, potatoes, and added sugars in foods and beverages than high-quality carbohydrates represented by whole grain, non-starch vegetables, legumes, and fruits. Protein intake from animal foods such as meat is still much higher than proteins from seafood and plant sources such as whole grains, nuts, and legumes. The consumption of saturated fat was above the Dietary Guidelines for Americans recommended level of 10% of energy intake and reached 11.6% in 2017–2018 cycles for type 2 diabetic adults [31]. Racial differences in diet quality as measured by HEI-2015 score might be related to multiple factors, including social economic status, education level, physical activities, etc. [38].

Though our trends of HEI-2015 scores of most dietary components for the type 2 diabetic adults are comparable to that of the general population [7,8], differences are also apparent for some components. Because of the glucose management in diabetic population, consumption of added sugar in type 2 diabetic adults as reflected by its higher HEI-2015 scores was significantly lower than that of general adult population [7]. Whole fruit consumption improved slightly in the general population (between 1999–2000 and 2015–2016), while that of the type 2 diabetes deteriorated significantly between 1999–2000 and 2017–2018 cycles.

Because fruits and vegetables are the primary sources of VC [20], decreasing intake of fruit and vegetables might have contributed to the increasing inadequacy of VC (Table 4). Because of the association of metformin intake and deficiency of VB12, increasing intake of metformin and decreasing intakes of meat and milk, which are the dietary sources of VB12, might all have contributed to the increasing inadequacy of VB12 in type 2 diabetes. Declining intake of beef and increasing intake of poultry meat might have contributed to the increasing inadequacy of iron intake in type 2 diabetes because of the higher heme irons in beef than in poultry meat [11]. Heme iron is two to three times more absorbable than non-heme iron found in plant-based and iron-fortified food [39]. However, because beef is also known to have relatively higher cholesterol and saturated fats than poultry meat, beef is recommended to be consumed only in moderation [40]. Loss of iron intake resulting from low beef consumption may need to be compensated from other sources for diabetic people. Declining consumption of fruit and vegetables might also have contributed to lower potassium intake. Adequate potassium intake is critical for moderating the prevalence of hypertension in the diabetic population [18]. However, because elevated eGFR in the diabetic population can result in unbalanced potassium, management of potassium in diabetic adults needs to be monitored [41].

From 1999 to 2018, the percentage of type 2 diabetic adults continued to rise in the US population [1] (Table 1). The estimated mean percentage of type 2 diabetes was about 94.3% of the US diabetic adults in this study. This proportion is in line with the estimation of 90–95% in recent years, 94.8% for type 2 among the total diabetes in 2017–2018, from US CDC for adult population of 20 years and older [42]. Interventions are needed to stabilize and possibly reverse the declining trends of vegetable and fruit consumption, and to further decrease the intake of refined grain and increase the intake of whole grain. In addition, efforts are still needed to discourage intake of saturated fat and added-sugar products to slow the growth of their shares in the total diet of the type 2 diabetic population. Supplements may also need to be studied for nutrients that are affected by the changing dietary patterns.

## 5. Limitations

There are several limitations in this study. Measurement errors from day-to-day variations in food intake are common in self-reported dietary 24 h recall data. Changes in dietary assessment methods over the study period may affect estimated accuracies in macronutrient intake across all cycles. Because cross-sectional data were used in the current study, causal inferences are not possible.

## 6. Conclusions

From 1999 to 2018, overall diet quality of US diabetic adults worsened based on the HEI-2015 scores. There were significant increases in consumption of saturated fats and added-sugar products, while consumptions of fruit and vegetables decreased significantly for type 2 diabetic adults. A significant improvement was made in reduced consumption of refined grains, and increased consumption of seafood and plant proteins and greens and beans. Despite the reduction in consumption of refined grain, low-quality carbohydrates and saturated fat remained high. The continued decline in consumption of fruit and vegetables might have worsened the intake inadequacy of vitamin C and potassium. Replacement of meat with poultry in diet might have contributed to the increased intake inadequacy of iron. In addition, declining intake of milk and meat and increased intake of metformin might also have contributed to the increased vitamin B12 inadequacy in US type 2 diabetic adults.

## Figures and Tables

**Table 1:** Demographic data and population means of BMI, A1c, and eGFR.

		1999–2000	2001–2002	2003–2004	2005–2006	2007–2008	2009–2010	2011–2012	2013–2014	2015–2016	2017–2018	^a^ *p* for Trend
Type 2 diabetes count	Total count	581	648	643	609	970	934	880	881	1014	1083	
	Men	290	331	327	306	485	491	450	446	535	566	
	Women	291	317	316	303	485	443	430	435	479	517	
	Mexican A	190	155	174	142	179	210	99	151	241	163	
	Hispanic	41	31	16	17	107	108	101	84	145	101	
	NH White	193	279	288	238	383	348	245	308	268	335	
	NH Black	136	155	138	190	272	210	299	217	230	265	
	Others	21	28	27	22	29	58	136	121	130	219	
Type 2 diabetes	% in total population (95% CI)	7.1%	8.0%	9.0%	9.0%	10.6%	10.5%	10.9%	11.4%	13.2%	13.5%	<0.001
		(6.2–8.2%)	(7.1–8.9%)	(7.8–10.4%)	(7.9–10.2%)	(9.4–12.0%)	(9.5–11.7%)	(9.7–12.1%)	(10.5–12.5%)	(11.6–15.0%)	(12.3–14.8%)	+
	Age (95% CI)	58.0	56.7	58.7	59.2	59.1	60.2	58.6	59.5	58.6	60.3	
		(56–60.1)	(54.7–58.8)	(56.5–60.8)	(57.3–61.2)	(58–60.2)	(58.5–61.8)	(57.4–59.8)	(58.4–60.6)	(57.9–59.3)	(58.4–62.1)	
	^b^ BMI (95% CI)	32.3	32.5	32.0	33.1	33.0	33.5	33.4	33.5	32.8	33.6	0.001
		(31.2–33.5)	(31.2–33.8)	(31.1–32.9)	(32.1–34)	(32.3–33.6)	(32.6–34.4)	(32.6–34.1)	(32.6–34.4)	(32.2–33.4)	(32.4–34.8)	+
	^c^ A1C (95% CI)	7.8	7.3	7.1	7.0	7.1	7.1	7.4	7.3	7.2	7.2	0.098
		(7.5–8)	(7.1–7.6)	(7–7.3)	(6.8–7.3)	(7–7.3)	(6.9–7.3)	(7.2–7.5)	(7.1–7.4)	(7–7.4)	(7–7.3)	+
	^d^ eGFR	83.8	86.4	83.7	82.1	84.1	82.0	84.0	82.2	85.4	84.1	0.001
	(95% CI)	(80.5–87.1)	(84.1–88.7)	(80.6–86.9)	(79.1–85.1)	(82.3–85.9)	(79.9–84.1)	(81.6–86.5)	(80.5–83.8)	(84.1–86.8)	(81.5–86.8)	+

^a^ *p*: significances of trend were adjusted for population weight, age, sex, and races; + indicates an increasing trend, and - a decreasing trend. Data in parentheses are 95% confidence intervals (CI). ^b^ BMI: body mass index (kg/m^2^). ^c^ A1C: hemoglobin A1C or HbA1C (%). ^d^ eGFR: estimated glomerular filtration rate (mL/min/1.73 m^2^). Other abbreviations: Mexican A, Mexican Americans; NH white, non-Hispanic white; NH black, non-Hispanic black.

**Table 2:** Estimated means, their 95% CI, and trends of the total and component scores of Healthy Eating Index (HEI)-2015 of the US type 2 diabetic adults in NHANES survey cycles 1999–2018.

	1999–2000	2001–2002	2003–2004	2005–2006	2007–2008	2009–2010	2011–2012	2013–2014	2015–2016	2017–2018	% Change from 1999–2000 to 2017–2018	^a^ *p*
Total HEI 2015 score	52.35	52.27	50.79	51.49	51.39	51.40	51.61	51.64	50.68	50.52	−3.5%	0.02
	50.8–53.9	49.9–54.7	49–52.6	50.2–52.8	50–52.8	50.2–52.6	49.8–53.4	50.4–52.9	49.7–51.6	49–52.1	−7.6–0.6%	-
**HEI-2015 scores and their 95% CI of adequacy components**												
Total vegetables (0–5)	3.35	3.28	3.07	3.43	3.29	3.08	3.30	3.15	3.22	2.98	−11.0%	0.017
	3.07–3.62	3.01–3.54	2.89–3.26	3.25–3.6	3.13–3.45	2.94–3.23	3.11–3.48	2.99–3.3	3.03–3.41	2.79–3.16	−20.1–2.0%	-
Greens and beans (0–5)	1.40	1.16	1.14	1.25	1.46	1.46	1.26	1.49	1.60	1.48	5.8%	0.038
	1.15–1.65	0.97–1.35	0.92–1.37	1.04–1.47	1.22–1.7	1.3–1.62	1.08–1.45	1.25–1.74	1.34–1.85	1.25–1.72	−19.3–30.8%	+
Total fruit (0–5)	2.71	2.50	2.42	2.39	2.24	2.34	2.09	2.02	2.00	2.16	−20.2%	<0.001
	2.4–3.01	2.15–2.86	2.1–2.74	2.2–2.57	2.03–2.45	2.12–2.57	1.93–2.24	1.84–2.2	1.8–2.21	1.94–2.38	−32.2–8.2%	-
Whole fruit (0–5)	3.31	2.94	2.96	2.38	2.31	2.27	2.09	2.14	2.15	2.34	−29.3%	<0.001
	3.03–3.59	2.55–3.34	2.62–3.3	2.22–2.55	2.07–2.56	2.04–2.5	1.85–2.34	1.95–2.33	1.89–2.41	2.07–2.61	−39.4–19.1%	-
Whole grains (0–5)	2.49	2.89	2.54	2.91	2.48	2.86	2.99	2.97	3.06	2.68	7.4%	0.355
	2.05–2.94	2.33–3.46	2.07–3.01	2.48–3.35	2.15–2.8	2.63–3.09	2.48–3.5	2.63–3.3	2.69–3.44	2.25–3.1	−18.0–32.8%	+
Total dairy (0–10)	4.66	4.64	4.49	5.11	4.89	5.19	5.00	4.97	4.97	4.36	−6.6%	0.899
	4.23–5.09	4.2–5.07	4.03–4.96	4.83–5.39	4.48–5.29	4.89–5.5	4.65–5.34	4.64–5.31	4.64–5.3	3.9–4.82	−19.5–6.4%	-
Total protein foods (0–5)	4.23	4.13	4.24	4.36	4.43	4.41	4.30	4.24	4.28	4.18	−0.9%	0.9
	4.1–4.34	3.87–4.38	4.05–4.42	4.21–4.51	4.36–4.51	4.31–4.5	4.19–4.4	4.13–4.36	4.12–4.44	4.04–4.33	−5.3–3.5%	-
Seafood & plant proteins (0–5)	1.71	1.85	2.03	2.05	2.21	2.34	2.16	2.47	2.31	2.40	39.7%	<0.001
	1.45–1.98	1.57–2.12	1.71–2.36	1.74–2.35	1.99–2.42	2.11–2.57	1.92–2.41	2.15–2.8	2.1–2.52	2.06–2.74	10.9–68.5%	+
Fatty Acids (0–10)	5.23	5.40	5.29	4.98	5.08	4.80	5.52	5.22	4.83	5.20	−0.5%	0.409
	4.68–5.77	4.64–6.16	4.74–5.83	4.55–5.4	4.62–5.54	4.48–5.13	5.17–5.87	4.8–5.64	4.4–5.25	4.75–5.64	−13.9–12.8%	-
**HEI-2015 scores and their 95% CI of moderation components**												
Sodium (0–10)	4.16	4.13	4.28	3.76	3.96	3.48	3.58	3.87	3.60	4.23	1.9%	0.284
	3.44–4.87	3.84–4.43	4.08–4.48	3.39–4.13	3.68–4.25	3.16–3.8	3.26–3.89	3.54–4.21	3.23–3.97	3.91–4.55	−17.1–21.0%	+
Refined grains (0–10)	5.46	5.50	5.22	5.83	6.00	5.86	5.84	6.06	6.08	5.84	7.0%	<0.001
	4.83–6.09	5–6	4.84–5.61	5.43–6.23	5.57–6.42	5.51–6.22	5.42–6.27	5.77–6.34	5.75–6.4	5.61–6.07	−6.0–20.0%	+
Saturated fats	6.06	6.50	5.56	5.32	5.61	5.80	5.85	5.72	5.26	5.41	−10.6%	0.001
(0–10)	5.56–6.56	5.86–7.13	5.08–6.04	4.84–5.8	5.16–6.05	5.49–6.11	5.52–6.17	5.35–6.08	4.96–5.56	4.99–5.84	−20.8–0.5%	-
Added sugars (0–10)	7.62	7.36	7.55	7.73	7.45	7.50	7.64	7.32	7.34	7.26	−4.5%	0.026
	7.1–8.1	6.8–7.9	7.1–8	7.4–8	7.1–7.8	7.2–7.8	7.2–8.1	7.1–7.5	7–7.7	7–7.5	−12.1–3.1%	-

^a^ *p*: significances of trend were adjusted for population weight, age, sex, and races.; + indicates an increasing trend, and - a decreasing trend. The number in the first row of a cell is the mean and numbers in the second row of the same cells are 95% CI. The numbers in the parentheses of the first column are the ranges of HEI-2015 score of the respective component.

**Table 3:** Estimated means, their 95% CI, and trends of major food components (categories) of US type 2 diabetic adults of NHANES survey cycles between 1999–2000 and 2017–2018.

	1999–2000	2001–2002	2003–2004	2005–2006	2007–2008	2009–2010	2011–2012	2013–2014	2015–2016	2017–2018	% Change from 1999–2000 to 2017–2018	^a^ *p*
**Fruits and vegetables (cup equivalent)**												
whole fruits	0.52	0.41	0.48	0.14	0.22	0.17	0.17	0.17	0.24	0.29	−45.3%	<0.001
	0.35–0.69	0.33–0.49	0.33–0.63	0.1–0.19	0.14–0.31	0.13–0.21	0.14–0.2	0.1–0.24	0.17–0.31	0.19–0.38	−71.1–19.5%	-
Total fruits	1.14	1.05	1.10	0.87	0.90	1.02	0.85	0.84	0.82	0.93	−18.2%	0.001
	0.92–1.36	0.89–1.2	0.84–1.37	0.75–0.98	0.78–1.03	0.87–1.17	0.77–0.92	0.76–0.93	0.7–0.94	0.81–1.06	−37.4–0.9%	-
dark vegetables	0.13	0.09	0.06	0.11	0.10	0.15	0.11	0.12	0.14	0.12	−10.8%	0.093
	0.06–0.2	0.07–0.1	0.04–0.09	0.07–0.16	0.07–0.13	0.1–0.19	0.07–0.16	0.09–0.15	0.1–0.19	0.09–0.14	−63.0–41.3%	-
Tomato & products	0.28	0.29	0.30	0.30	0.27	0.25	0.35	0.27	0.30	0.26	−8.0%	0.593
	0.22–0.35	0.25–0.34	0.23–0.37	0.23–0.38	0.24–0.31	0.21–0.28	0.28–0.41	0.24–0.3	0.25–0.34	0.22–0.3	−33.8–17.8%	-
Potatoes	0.38	0.46	0.43	0.36	0.35	0.35	0.44	0.36	0.36	0.36	−4.3%	0.172
	0.31–0.44	0.38–0.54	0.35–0.52	0.3–0.42	0.3–0.4	0.3–0.4	0.33–0.55	0.3–0.41	0.31–0.42	0.31–0.41	−25.6–16.9%	-
total vegetables	1.64	1.64	1.52	1.63	1.48	1.46	1.74	1.47	1.51	1.42	−13.7%	0.077
	1.37–1.91	1.45–1.84	1.37–1.67	1.49–1.76	1.38–1.58	1.34–1.59	1.5–1.97	1.37–1.57	1.37–1.64	1.26–1.57	−30.7–3.3%	-
**Grains, nuts, and seeds (ounce equivalent)**												
Whole grains	0.72	0.93	0.82	0.84	0.65	0.92	1.01	0.97	1.02	0.88	21.7%	0.080
	0.58–0.86	0.68–1.18	0.63–1.01	0.69–0.99	0.56–0.74	0.79–1.06	0.75–1.27	0.84–1.1	0.84–1.19	0.69–1.06	−12.9–56.4%	+
Refined grains	5.42	5.57	5.92	5.12	5.11	5.46	5.60	5.29	5.21	5.43	0.2%	0.310
	4.86–5.98	4.83–6.31	5.39–6.44	4.75–5.5	4.75–5.48	5.15–5.77	5.07–6.13	5.09–5.5	4.85–5.58	5.01–5.86	−12.7–13.2%	+
Total grains	6.14	6.50	6.73	5.96	5.76	6.39	6.61	6.26	6.23	6.31	2.7%	0.818
	5.56–6.72	5.56–7.45	6.13–7.33	5.58–6.35	5.39–6.13	6.07–6.7	6.03–7.19	6.01–6.51	5.84–6.62	5.82–6.79	−9.7–15.2%	+
Nuts and seeds	0.39	0.46	0.71	0.51	0.60	0.54	0.63	0.71	0.63	0.77	54.4%	0.001
	0.23–0.54	0.31–0.6	0.48–0.93	0.36–0.65	0.46–0.74	0.44–0.65	0.49–0.77	0.57–0.85	0.46–0.79	0.65–0.89	18.8–89.9%	+
**Meat and fish (ounce equivalent)**												
meat	1.75	1.94	1.87	1.49	1.60	1.64	1.59	1.34	1.64	1.41	−19.7%	0.003
	1.52–1.98	1.59–2.29	1.55–2.18	1.23–1.74	1.41–1.78	1.45–1.84	1.27–1.9	1.17–1.52	1.4–1.89	1.15–1.66	−37.7–1.8%	-
Poultry	1.50	1.14	1.14	1.39	1.46	1.36	1.42	1.36	1.14	1.53	2.0%	0.409
	1.15–1.85	0.9–1.39	0.79–1.49	1.13–1.64	1.21–1.71	1.08–1.64	1.12–1.72	1.17–1.55	0.93–1.35	1.27–1.78	−27.1–31.1%	+
seafood	0.79	0.76	0.84	0.53	0.48	0.81	0.60	0.75	0.64	0.67	−15.7%	0.501
	0.43–1.16	0.55–0.96	0.54–1.13	0.35–0.71	0.36–0.59	0.54–1.07	0.36–0.83	0.54–0.96	0.28–1	0.45–0.89	−63.0–31.5%	-
Total meat	4.79	4.63	4.73	4.66	4.68	5.19	4.62	4.46	4.58	4.61	−3.8%	0.617
	4.32–5.26	4.17–5.09	4.14–5.32	4.16–5.16	4.31–5.05	4.83–5.54	4.21–5.04	4.08–4.83	3.9–5.27	4.16–5.05	−16.9–9.3%	-
**Eggs (Ounce equivalents) and dairy (cups equivalents)**												
Eggs	0.54	0.58	0.56	0.59	0.54	0.54	0.66	0.57	0.64	0.64	18.0%	0.180
	0.37–0.71	0.46–0.7	0.48–0.63	0.43–0.76	0.47–0.61	0.46–0.62	0.55–0.77	0.43–0.7	0.54–0.75	0.52–0.76	−25.5–61.6%	+
Milk	0.81	0.82	0.82	0.79	0.78	0.86	0.75	0.68	0.73	0.53	−34.9%	<0.001
	0.68–0.94	0.67–0.97	0.66–0.98	0.69–0.9	0.67–0.9	0.74–0.98	0.58–0.92	0.57–0.8	0.6–0.87	0.44–0.62	−50.1–19.8%	-
Cheese	0.41	0.44	0.52	0.50	0.48	0.61	0.68	0.71	0.60	0.61	49.9%	<0.001
	0.29–0.53	0.34–0.54	0.35–0.7	0.4–0.6	0.38–0.58	0.54–0.68	0.58–0.78	0.62–0.81	0.53–0.68	0.53–0.69	2.7–97.0%	+
Total dairy	1.24	1.31	1.36	1.35	1.33	1.53	1.51	1.48	1.43	1.21	−1.9%	0.295
	1.1–1.3	1.1–1.5	1.1–1.6	1.2–1.5	1.2–1.5	1.4–1.7	1.3–1.7	1.3–1.6	1.3–1.6	1.1–1.4	−16.1–12.2%	-
**Other macronutrients and energy**												
Oils (grams)	13.60	16.18	18.96	20.00	19.64	20.06	24.65	25.14	26.16	27.29	100.6%	<0.001
	11.2–16	13.8–18.6	16.3–21.6	18.2–21.8	17.9–21.4	18.1–22	22.8–26.5	23.6–26.7	23.4–28.9	25–29.6	61.8–139.4%	+
Solid Fats (grams)	42.53	40.02	44.61	38.47	36.24	37.57	36.84	33.24	33.60	33.90	−20.3%	<0.001
	38.1–47	35.4–44.6	40.3–48.9	34.2–42.7	33.4–39.1	35.6–39.5	33.9–39.8	30.3–36.2	31–36.2	31.2–36.6	−30.8–9.8%	-
Added Sugars (tsp. Equivalent)	13.38	13.28	12.91	11.84	13.25	13.83	12.79	13.84	14.13	14.17	5.9%	0.031
	10.9–15.9	11–15.5	10.9–14.9	10.4–13.3	11.5–15	12.5–15.2	11.3–14.3	12.5–15.2	12.6–15.7	12.6–15.8	−17.1–28.9%	+
Energy (kcal)	1837	1894	1991	1844	1843	1955	1987	1920	1944	1943	5.7%	0.058
	1718–1956	1786–2002	1856–2126	1725–1962	1757–1929	1873–2038	1890–2083	1855–1985	1814–2075	1832–2055	−3.3–14.9%	+
^b^ Sfat/energy percent	10.8%	10.4%	11.6%	12%	11.4%	11.3%	11.2%	11.3%	11.8%	11.6%	7.7%	+
	10.3–11.3%	9.6–11.1%	11–12.1%	11.4–12.5%	11–11.9%	11–11.6%	10.9–11.4%	10.8–11.7%	11.4–12.2%	11.1–12.1%	0.7%–14.6%	0.03

^a^ *p*: significance levels were adjusted for population weight, age, sex, and races; <0.05 indicates significant with 95 confidence level (CI), + indicates an increasing trend, and - a decreasing trend and *p* calculations were adjusted for age, gender, and race. Only 21 major food components/categories (with equivalent amount > 0.1) of the 37 components/categories and the total energy were listed here. ^b^ Sfat/energy percent: saturated fat as percent of total energy. The number in the first row of a cell is the mean and numbers in the second row of the same cell are the 95% CI.

**Table 4:** Estimated medians and percentages of inadequacies of usual intakes of VC, VB12, Fe, and K for US type 2 diabetic adults between sample years 2003–2004 and 2017–2020.

	2003–2004	2005–2006	2007–2008	2009–2010	2011–2012	2013–2014	2015–2016	2017–2018	2017–2020	^a^ *p*
Estimated median (and standard errors) of usual intake of dietary + supplement intake (mg/24 h)										
VC	94.2 (8.3)	89.9 (3.9)	85.2 (7)	87.8 (5)	88.5 (5.3)	87.2 (4.8)	90.7 (6.4)	87.7 (6.2)	89.7 (4.2)	<0.01 -
VB12	5.5 (0.4)	5.7 (0.4)	5.5 (0.2)	6.4 (0.3)	5.9 (0.5)	5.2 (0.3)	5.6 (0.6)	6 (0.5)	6.2 (0.3)	<0.01 +
Fe	14.9 (0.7)	14.9 (0.4)	14.3 (0.5)	15.5 (0.6)	15.1 (0.6)	14.4 (0.4)	14.6 (0.6)	14 (0.5)	13.5 (0.4)	<0.01 -
K	2614 (76)	2466 (88)	2444 (51)	2547 (74)	2654 (144)	2501 (30)	2517 (79)	2438 (88)	2428 (64)	<0.01 -
Estimated median (and standard errors) of usual intake of dietary intake only (mg/24 h)										
VC	73.4 (7)	66.2 (2.8)	63.7 (3.7)	68.4 (3.2)	69.6 (4.1)	59 (2.7)	60.7 (3.7)	62.7 (3.9)	62.3 (3.1)	<0.01 -
VB12	4.8 (0.14)	5.2 (0.11)	4.9 (0.14)	5 (0.08)	4.9 (0.1)	4.6 (0.11)	4.6 (0.12)	4.5 (0.12)	4.3 (0.08)	<0.01 -
Fe	14.1 (0.55)	14.3 (0.36)	13.7 (0.43)	14.4 (0.45)	14.5 (0.57)	13.3 (0.29)	13.6 (0.52)	12.9 (0.45)	12.7 (0.32)	<0.01 -
K	2517 (82)	2428 (89)	2430 (59)	2536 (73)	2650 (136)	2485 (43)	2503 (87)	2450 (94)	2421 (68)	<0.01 -
Percent (%) of inadequate intake (and standard error) with dietary and supplement intake										
VC	34.6 (4.4)	35 (2.6)	39.6 (3.5)	37.1 (3.1)	35.2 (4.2)	35.8 (3.3)	37.2 (3.1)	36.5 (4)	36.8 (2.3)	<0.01 +
VB12	2.9 (1.11)	2.2 (1.75)	1.5 (0.79)	1.4 (0.8)	3.4 (1.16)	1.5 (0.96)	2.3 (2.84)	5.7 (1.35)	4.4 (0.8)	<0.01 +
Fe	0.8 (0.61)	0.8 (0.41)	1.3 (0.52)	1.5 (0.45)	1.1 (0.57)	1.4 (0.85)	1.9 (0.76)	2.8 (0.9)	2.7 (0.61)	<0.01 +
K	64.4 (3.2)	69.9 (3.2)	74.3 (2.4)	70 (2.9)	65.4 (6.4)	70.2 (2.2)	70.1 (3.5)	73.2 (2.3)	74.2 (2.2)	<0.01 +
Percent (%) of inadequate intake (and standard error) with only dietary intake										
VC	44.5 (5.8)	50.2 (2.8)	53.7 (3.6)	49.2 (2.9)	47.6 (4.3)	58.9 (2.9)	56.8 (3.5)	54.8 (3.6)	55.1 (3.1)	<0.01 +
VB12	3 (0.72)	2.8 (0.53)	2.6 (0.66)	1.9 (0.34)	3.3 (0.73)	3.8 (0.92)	4.1 (0.68)	6 (1.05)	6.1 (0.78)	<0.01 +
Fe	0.9 (0.65)	0.8 (0.45)	1.4 (0.41)	1.6 (0.48)	1.3 (0.57)	2.1 (1)	2.5 (1.08)	3.1 (0.87)	2.9 (0.69)	<0.01 +
K	67.5 (3)	72.4 (3.4)	75.6 (2.1)	70.3 (2.8)	65.5 (5.7)	71.2 (1.8)	71.1 (3.7)	72.6 (2.9)	74.4 (2.6)	<0.01 +

^a^ *p*: significances of trend were adjusted for population weight, age, sex, and races; <0.05 indicates significant with 95 confidence level, + indicates an increasing trend, and - indicates a decreasing trend. Numbers in the parentheses are standard errors.

## Data Availability

All data for this study is publicly available on the National Center for Health Statistics websites, available at: https://wwwn.cdc.gov/nchs/nhanes/search/datapage.aspx (all accessed on 4 April 2023).

## Supplementary Materials

The following supporting information can be downloaded at: https://www.mdpi.com/article/10.3390/nu15081980/s1, Table S1: Standards for scoring of dietary components of Healthy Eating Index-2015; Table S2: Definition of major categories of dietary components by food sources (from USDA).
