# Supplementary material for: Trends in Diet Quality and Increasing Inadequacies of Micronutrients Vitamin C, Vitamin B12, Iron and Potassium in US Type 2 Diabetic Adults
Source: Nutrients. 2023 Apr 20;15(8):1980. doi: 10.3390/nu15081980 (PMC10142510; doi:10.3390/nu15081980)
Supplement: Supplementary file 1 [file nutrients-15-01980-s001.zip › nutrients-2299656-supplementary.pdf]

Supplemental Tables (s1 to s2)

Table s1. Standards for scoring of dietary components of Healthy Eating Index-2015

	Maximum points	standard for maximum score	Standard for minimum score of zero
Adequacy Component			
Total fruit	5	≥0.8 [a]c equivalents/1000 kcal	No fruit
Whole fruit	5	≥0.4 c equivalents/1000 kcal	No whole fruit
Total vegetables	5	≥1.1 c equivalents/1000 kcal	No vegetables
Greens and beans	5	≥0.2 c equivalents/1000 kcal	No dark green vegetables or beans and peas
Whole grains	10	≥1.5 [b]oz equivalents/1000 kcal	No whole grains
Total dairy	10	≥1.3 c equivalents/1000 kcal	No dairy
Total protein foods	5	≥2.5 oz equivalents/1000 kcal	No protein foods
Seafood and plant proteins	5	≥0.8 c equivalents/1000 kcal	No seafood or plant
Fatty Acids	10	[c](PUFAs+MUFAs) /SFAs≥2.5	(PUFAs+MUFAs)/SFAs≤1.2
Moderation Component			
Sodium	10	≤1.8 oz equivalents/1000 kcal	≥4.3 oz equivalents/1000 kcal
Refined grains	10	≤1.1 g/1000 kcal	≥2.0 g/1000 kcal
Saturated fats	10	≤6.5% of energy	≥26% of energy
Added sugars	10	≤8% of energy	≥16% of energy

Abbreviation: [a]c :cups, [b]oz: ounces, [c]PUFAs and MUFAs, poly-and monosaturated fatty acids; SFAs, saturated fatty acids

Table s2. Definition of major categories of dietary components by food sources (from USDA)[a]

Category	Items
Whole Fruits	Intact fruits (whole or cut) of citrus, melons, and berries
Total fruits	Total intact fruits (whole or cut) and fruit juices
Dark Vegetables	Dark green vegetables
Tomato and products	Tomatoes and tomato products
Potatoes	White potatoes (cup eq.)
Total Vegetables	Total dark green, red and orange, starchy, and other vegetables; excludes legumes
Whole grain	Whole-wheat flour, bulgur (cracked wheat), whole barley, whole-barley flour, oatmeal, oats, rye, whole cornmeal, popcorn, wild rice, brown rice, etc.
Refined grain	White flour, de-germed cornmeal, white bread, white rice, etc.
Total grain	Total whole and refined grains
Nuts and Seeds	Peanuts, tree nuts, and seeds; excludes coconut
Meat	Beef, veal, pork, lamb, and game meat; excludes organ meat and cured meat
Poultry	Chicken, turkey, Cornish hens, duck, goose, quail, and pheasant (game birds); excludes organ meat and cured meat
Seafood	Finfish, shellfish, and other seafood
Total meat	Total of meat, poultry, seafood, organ meat, and cured meat
Egg	Eggs; egg whites or egg beaters; omega-3 fortified eggs; mayonnaise
Milk	Skim milk; 1 or 2 milk; whole milk
Cheese	cottage or ricotta cheese; other cheese e.g. American, cheddar, etc.
Total Dairy	Total milk, yogurt, cheese, and whey.
Oils	Fats naturally present in nuts, seeds, and seafood; all un-hydrogenated vegetable oils, except palm oil, palm kernel oil, and coconut oils; the fat present in avocado and olives above the allowable amount; 50% of the fat present in stick and tub margarines and margarine spreads (grams)
Solid fats	Fats naturally present in meat, poultry, eggs, and dairy (lard, tallow, and butter); fully or partially hydrogenated oils; shortening; palm oil; palm kernel oil; coconut oils; fats naturally present in coconut meat and cocoa butter; and 50 of the fat present in stick and tub margarines and margarine spreads (grams)

Added sugars	Caloric sweeteners such as syrups and sugars and others

[a]Note: definitions are adapted from https://www.ars.usda.gov/ARSUserFiles/80400530/pdf/fped/FPED_1718.pdf
